# Specialised teams or personal continuity across inpatient and outpatient mental healthcare? Study protocol for a natural experiment

**DOI:** 10.1136/bmjopen-2015-008996

**Published:** 2015-11-25

**Authors:** Domenico Giacco, Victoria Jane Bird, Paul McCrone, Vincent Lorant, Pablo Nicaise, Andrea Pfennig, Michael Bauer, Mirella Ruggeri, Antonio Lasalvia, Jacek Moskalewicz, Marta Welbel, Stefan Priebe

**Affiliations:** 1 Unit for Social and Community Psychiatry (World Health Organisation Collaborating Centre for Mental Health Services Development), Queen Mary University of London, London, UK; 2 Health Service and Population Research Department, Centre for the Economics of Mental and Physical Health, Institute of Psychiatry, Psychology and Neuroscience, King's College London, London, UK; 3 Institute of Health and Society IRSS, Université catholique de Louvain, Bruxelles, Belgium; 4 Department of Psychiatry and Psychotherapy, Carl Gustav Carus University Hospital, Technische Universität Dresden, Dresden, Germany; 5 Section of Psychiatry, Department of Public Health and Community Medicine, University of Verona, Verona, Italy; 6 Institute of Psychiatry and Neurology, Warsaw, Poland

**Keywords:** MENTAL HEALTH, PUBLIC HEALTH

## Abstract

**Introduction:**

Mental healthcare organisation can either pursue *specialisation*, that is, distinct clinicians and teams for inpatient and outpatient care or *personal continuity of care*, that is, the same primary clinician for a patient across the two settings. Little systematic research has compared these approaches. Existing studies subject have serious methodological shortcomings. Yet, costly reorganisations of services have been carried out in different European countries, inconsistently aiming to achieve specialisation or personal continuity of care. More reliable evidence is required on whether specialisation or continuity of care is more effective and cost-effective, and whether this varies for different patient groups and contexts.

**Design and methods:**

In a natural experiment, we aim to recruit at least 6000 patients consecutively admitted to inpatient psychiatric care in Belgium, Germany, Italy, Poland, and the UK. In each country, care approaches supporting specialisation and personal continuity coexist. Patients will be followed up at 1 year to compare outcomes, costs and experiences. Inclusion criteria are: 18 years of age or older; clinical diagnosis of psychosis, affective disorder or anxiety/somatisation disorder; sufficient command of the language of the host country; absence of cognitive deterioration and/or organic brain disorders; and capacity to provide informed consent.

**Ethics and dissemination:**

Ethical approval was obtained in all countries: (1) England: NRES Committee North East—Newcastle & North Tyneside (ref: 14/NE/1017); (2) Belgium: Comité d'Ethique hospitalo-facultaire des Cliniques St-Luc; (3) Germany: Ethical Board, Technische Universität Dresden; (4) Italy: Comitati Etici per la sperimentazione clinica (CESC) delle provincie di Verona, Rovigo, Vicenza, Treviso, Padova; (5) Poland: Komisja Bioetyczna przy Instytucie Psychiatrii i Neurologii w Warszawie. We will disseminate the findings through scientific publications and a study-specific website. At the end of the study, we will develop recommendations for policy decision-making, and organise national and international workshops with stakeholders.

**Trial registration number:**

ISRCTN registry: ISRCTN40256812.

**Strengths and limitations of this study**

- This study will be the largest one comparing care approaches based on specialisation and personal continuity of care worldwide.
- It will involve services across five European countries and comprehensively assess outcomes, costs and experiences of the ‘specialisation’ and ‘personal continuity’ care approaches.
- The natural experiment methodology has the advantage of allowing comparisons of the two approaches without altering already established routine provision of care.
- Being a natural experiment, patients will not be randomised to either approach. Clustering in outcomes may be found within hospitals and countries. We will explore random effects for hospitals using mixed regression models.
- Selection bias at baseline and attrition at follow-up are common pitfalls in large prospective studies on people with severe mental disorders. We will attempt to: (1) minimise selection bias through approaching all patients within a few days from a hospital admission; and (2) reduce attrition using medical records or brief phone interviews to collect the main outcome data.

## Introduction

A central question in mental healthcare is whether to prioritise specialisation of clinicians and clinical teams or personal continuity of care.^1–4^ These two care approaches are sometimes incompatible as each one requires a different organisation of care.^1^ ^5^

Following the specialisation approach, mental healthcare should be provided by different clinicians and teams in inpatient and outpatient settings. This care approach is expected to simplify the practical organisation of services; support quick clinical decision-making; enable clinical teams and clinicians to have a full focus on only one setting; and foster an expertise in setting specific aspects of treatment.^1^ ^6^

According to the personal continuity of care approach, however, the same primary clinician is responsible for a given patient within both inpatient and outpatient settings. This approach may facilitate the smooth transition of patients from one setting to another; support long-lasting therapeutic relationships; and simplify clinical communication as patients and clinicians are familiar with each other across the care settings.^2^ ^3^ ^7^

The expected benefits of specialisation and personal continuity of care are summarised in table 1.

**Table 1:** Expected benefits of specialisation and personal continuity of care

Areas	Specialisation	Personal continuity of care
Organisational aspects at a service level	Quick clinical decision-making; positive risk management	No fragmentation of services; increased engagement with patients who are less likely to actively seek treatment
Clinical benefits	Specialisation of interventions	Continuity of care
Impact on routine care	Enhancement of clinical leadership and specialised expertise	Establishment of a stronger therapeutic relationship

Yet, solid evidence on which of these two approaches is associated with better outcomes is lacking. As a consequence, service reorganisations are inconsistent across Europe and the issue repeatedly raises debate.^3^ ^8–10^

In the UK, although the organisation of mental healthcare traditionally favoured personal continuity of care,^3^ ^7^ most provider organisations of mental healthcare (ie, NHS Trusts) have now established specialised hospital and community teams.^5^ ^11^ ^12^

In Germany, the traditional approach has been characterised by specialisation of clinicians and teams, while recent initiatives (‘Integrierte Versorgung’, ie, ‘Integrated healthcare’) have aimed to strengthen continuity of care and to make one service responsible for both inpatient and outpatient care.^2^ ^4^

**Figure 1:**
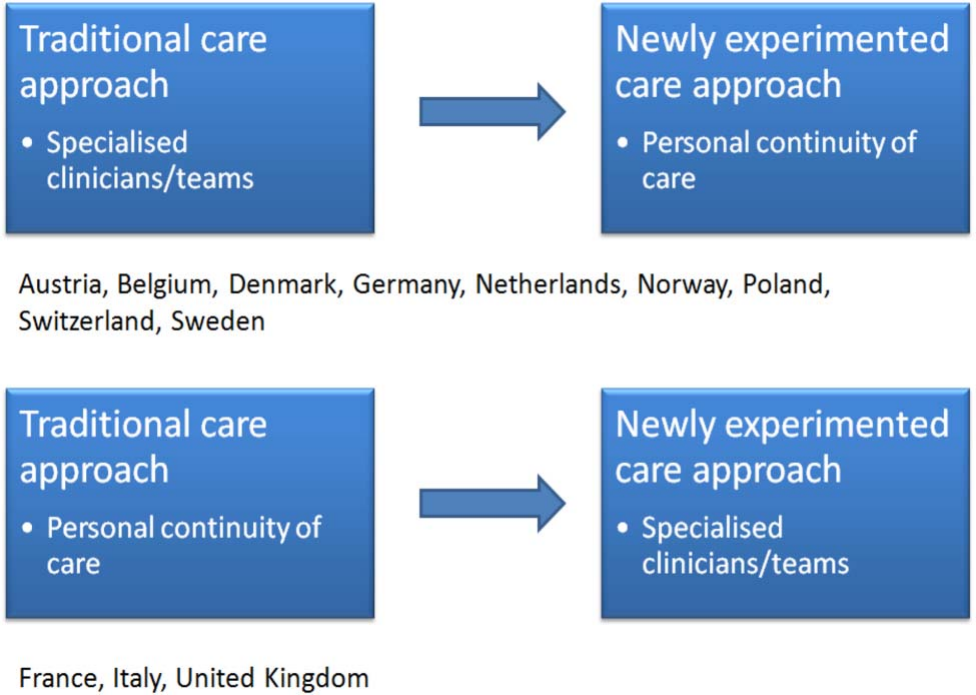
National reforms and changes in mental healthcare organisation.

In various other European countries (eg, Austria, Belgium, Denmark, France, Italy, Netherlands, Norway, Poland, Sweden, Switzerland) similar experimental programmes and professional debates are currently ongoing to change traditional mental healthcare approaches^13–20^ (see figure 1).

A systematic review comparing approaches based on specialisation or personal continuity of care found there was a general tendency towards better outcomes and patient/clinician preferences for approaches prioritising personal continuity of care.^21^ However, most studies had serious methodological shortcomings and definitive conclusions could not be drawn.

The studies included in the review shared the following limitations:^21^

- They were conducted in local settings.
- Their samples were not large enough to detect small differences in outcomes between care approaches. Yet, small differences, for example a reduction of 5% in the number of re-hospitalisations, may have a significant impact on a national scale.
- The studies have assessed only a limited range of outcomes, disregarding outcomes like patient safety and cost-effectiveness.
- All the studies compared a newly implemented care approach (either favouring specialisation, eg, in the UK or personal continuity of care, eg, in Germany) with the traditional approach. With the exception of patient experiences and preferences, which were consistently in favour of care approaches supporting personal continuity of care,^5^ ^22^ other outcomes were always positive for the new experimental approach irrespective of the type (‘novelty bias’).^21^

To address the limitations of the available studies, we have designed a natural experiment comparing approaches favouring either specialisation or personal continuity of care in five European countries (Belgium, Germany, Italy, Poland and UK). In all these countries, both approaches to care exist within routine practice.

Mental healthcare approaches were categorised based on whether a patient has the same ‘primary clinician’ for their inpatient and outpatient care or not. The ‘primary clinician’ is the clinician who is primarily responsible for the patient (usually a psychiatrist or a psychologist). The two approaches were operationalised as follows:

- ‘Specialisation approaches’ are those in which different primary clinicians are responsible for the treatment of a patient, depending on whether the patient is in the inpatient or outpatient care.
- ‘Personal continuity approaches’ are those in which the same primary clinician is responsible for both inpatient and outpatient care of a patient.

This study was funded by the 7th Framework Programme of the European Commission (Full title: Comparing policy, framework, structure, effectiveness and cost-effectiveness of functional and integrated systems of mental health care—COFI).

COFI will be the largest study comparing specialisation and personal continuity of care, reaching more patients than all of the previous studies combined. The study will involve a comprehensive assessment of outcomes, cost-effectiveness and patient/clinician experiences. It will include services in which specialisation or personal continuity approaches are the standard way of providing mental healthcare, rather than experimental programmes.

The comparison of the two approaches will be carried out in a wide range of settings, and in countries with different traditions and practices of mental healthcare. ‘Specialisation’ and ‘personal continuity’ approaches are supported in these different countries by a variety of funding mechanisms, policies and clinical arrangements. This will allow for the generalisability of our findings from the participating countries to a higher number of countries with similar characteristics.

## Aims and objectives

COFI aims to compare outcomes, costs and experiences of ‘specialisation’ and ‘personal continuity’ of care approaches.

Readmission to hospital was chosen as the primary outcome of the study due to the high clinical relevance and impact on service costs. The validity of readmission rates as an outcome criterion for mental health service evaluation has been confirmed in previous studies.^23^ ^24^

COFI will address the following research questions:

- Primary research question
  - Do rates of rehospitalisation differ between the specialisation and personal continuity approaches?
- Clinical and social outcomes
  - Is there a difference in number of voluntary and compulsory rehospitalisations per patient and yearly inpatient bed days between the two approaches?
  - Are untoward incidents (deaths, suicides, physical violence committed or experienced by patients) more frequent in either approach?
  - What are the social outcomes, in terms of employment, accommodation, living situation, subjective quality of life and social contacts of patients treated by either approach?
- Costs of care:
  - What are the direct costs of care (ie, related to use of services) within each approach?
  - Which approach is most cost-effective?
- Experience of care
  - What are treatment satisfaction and experiences of care provided by the two approaches and do they differ?

We will also carry out comparisons of the two approaches in subgroups of patients defined by age, gender, diagnosis, first admission, socioeconomic situation, migrant status and presence of physical comorbidities.

We hypothesise that:

- The ‘specialisation approach’ may be more effective in providing specialised care for people with less complex and more specific mental health needs (eg, absence of comorbid disorders or social problems), who do not require coordination of different interventions.^1^ ^12^
- The ‘personal continuity approach’, ensuring a stronger coordination of different interventions,^7^ ^25^ may be more suited for treating patients who are affected by severely disabling mental health conditions (eg, schizophrenia) and comorbid physical illnesses, or those who have complex psychosocial needs such as patients with a low socioeconomic status, migrants and patients with comorbidities.

## Design and methods

### Study design

COFI is a natural experiment, prospectively following up for 1 year patients who, at the point of entry in the study, are hospitalised within care services adopting either a ‘specialisation’ or ‘personal continuity’ approach. Participating sites are located in five countries (Belgium, Germany, Italy, Poland and UK). In these countries both approaches are present, although practice, traditions, policies and funding of mental healthcare vary significantly.

Each included patient can only be treated within either a ‘specialisation’ or ‘personal continuity’ care approach, and the exposure of patients to either approach is outside of the control of investigators and hence, naturalistic. Allocation to either model is based on the given organisation of care in a defined geographical area (UK, Italy), on a clinical decision and patients’ choice (Belgium, Poland), or on insurance arrangements (Germany).

The methodology of a natural experiment has the main advantage of allowing comparisons of the two approaches without altering already established routine provision of care.

However, this implies that no randomised allocation of patients to different approaches is possible. Given the risk of bias associated with a non-randomised design, adjustment for potential confounders is of paramount importance.^26^ In our study, potential confounders will be adjusted for by exploring random effects at a hospital level which will provide an estimate of the potential variability of practice and outcomes both within countries and in different services within the countries.

### Settings

COFI is coordinated by the Unit for Social and Community Psychiatry, Queen Mary University of London (QMUL). QMUL is responsible for data collection in 11 provider organisations, that is, National Health Service (NHS) Trusts, in the UK as well as for overall project management.

Collaborating academic centres which are responsible for data collection in the remaining four countries are: (1) University of Louvain (Belgium); (2) Technische Universität Dresden (Germany); (3) University of Verona (Italy); (4) Institute of Psychiatry and Neurology of Warsaw (Poland). In each country, at least four hospitals in which a ‘specialisation approach’ is used and four hospitals in which a ‘personal continuity approach’ is used will be included in data collection. In Belgium, Germany and Poland, patients treated under ‘specialisation’ and ‘personal continuity’ of care approaches can be admitted to the same hospital as organisation of care is based on insurance arrangements or clinical decisions for the individual patients.

**Figure 2:**
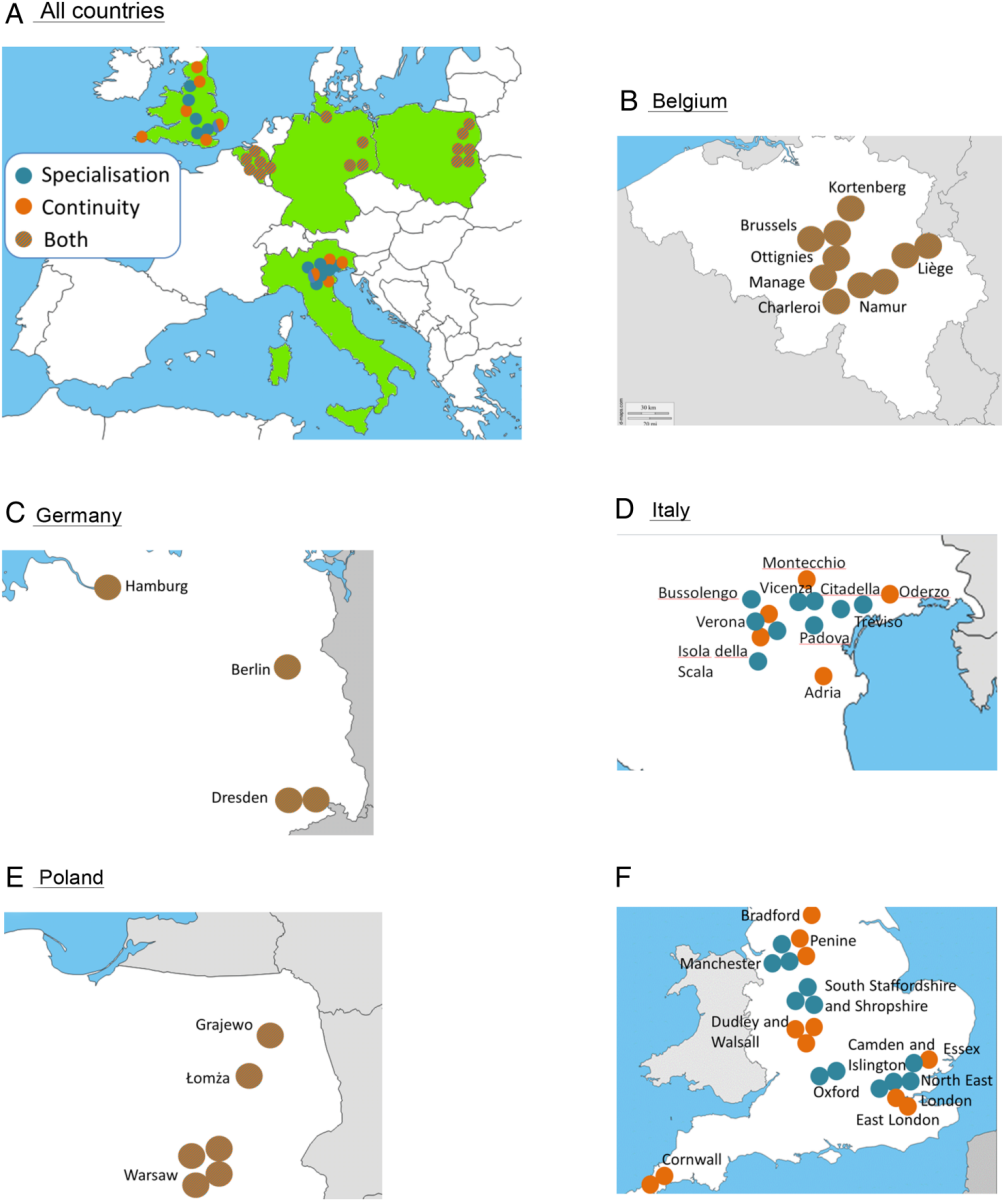
Hospitals included in the study.

The hospitals in which patients are recruited are spread throughout the participating countries (see figure 2).

.

### Participants

#### Inclusion criteria

(1) 18 years of age or older; (2) clinical diagnosis of psychotic disorder (F20–29), affective disorder (F30–39) or anxiety/somatisation disorder (F40–49) (International Classification of Diseases—ICD—10);^27^ (3) being hospitalised in a general adult psychiatric inpatient unit; (4) sufficient command of the language of the host country to provide written informed consent and understand the questions in the research interviews; (5) capacity to provide informed consent.

#### Exclusion criteria

(1) Diagnosis of organic brain disorders; (2) too severe cognitive impairment for providing information on the study instruments.

### Baseline measures

At baseline, we will collect data on:

- Sociodemographic characteristics: year of birth, gender, marital status, highest completed education level, country of birth;
- Social situation: employment, accommodation, living situation, friendships, self-reported benefits receipt due to low income;
- Psychiatric and non-psychiatric diagnoses according to the International Classification of Disease 10 (ICD-10);^27^ the primary clinical diagnosis is established from medical records or clinician’s report at discharge from hospital;
- Severity of illness, rated by treating psychiatrists by the Clinical Global Impression Scale (CGI);^28^
- Whether the current admission is the first one or not;
- Formal status at admission (involuntary/voluntary);
- Patient appraisal of inpatient care, as measured by the Client Assessment of Treatment (CAT).^29^

### Follow-up measures

In the one-year follow-up, we will compare ‘specialisation’ and ‘personal continuity’ of care approaches with reference to:

#### Primary outcome

- Readmission to hospital.

#### Other clinical and social outcomes

- *Clinical outcomes* including: (1) number of readmissions; (2) number of compulsory readmissions; (3) inpatient bed days, using an ad hoc schedule based on the Client Service Receipt Inventory.^30^
- *Social outcomes,* based on the SIX index,^31^ which captures*:* (1) employment (none; voluntary or protected or sheltered work; regular employment); (2) accommodation (homeless or 24 h supervised; sheltered or supported accommodation; independent accommodation); (3) living situation (living alone; living with a partner or family); (4) contacts with friends (not having met a friend within the past week; having met at least one friend in the past week).
- *Subjective quality of life*, measured by the Manchester Short Assessment of quality of life MANSA.^32^
- *Untoward incidents related to mental healthcare*, including: (a) deaths; (b) completed suicides; (c) serious assaults committed by patients; (d) physical violence experienced by patients; (e) suicide attempts; (f) serious side effects from pharmacological treatment requiring hospitalisation, as extracted from clinical records or obtained through phone interviews.
- *Social contacts,* measured as: (a) the total number of social contacts within the week preceding the assessment as reported by patients using an ad hoc instrument, adapted from the Social Network Schedule,^33^ and (b) whether or not they declare to have at least one close friend, using the specific item on the MANSA.^32^
- *Experienced discrimination,* measured as*:* (a) patient-reported episodes of discrimination because of mental illness, and (b) patient-reported avoidance of activities because of fear of discrimination, measured by ad hoc items.
- *Perceived socioeconomic status,* that is, patient self-rated standing in his/her own self-defined community and country, measured by the MacArthur Scale of Subjective Social Status.^34^ ^35^

#### Costs

Service use will be measured using an adapted version of the Client Service Receipt Inventory.^30^ This will include details of:

- In-patient bed days;
- Contacts with outpatient services;
- Contacts with day centres;
- Contacts with other community services.

We will then calculate unit costs for each service and for the different countries in order to translate service use data into costs of care.

#### Experience of care

- *Satisfaction with care*, measured by the 32-item version of the Verona Service Satisfaction Scale (VSSS-32)*.*^36^
- *Experienced continuity of care*, explored by a set of questions designed during the study preparation phase. The questions explore: a) whether patients, following discharge, have met any of the psychiatrists or other mental health professionals that they saw in hospital; b) if yes how many psychiatrists or mental health professionals and what type of professionals; c) how long was it in weeks between hospital discharge and first outpatient contact; and d) whether they feel this duration was too long, too short or just right.

Qualitative semistructured interviews will explore the in-depth experience of providing care (clinicians) and receiving care (patients) within specialisation or continuity of care approaches. Topic guides are being developed to constitute a discussion guide for the interviews. These are based on the same set of themes and topics for patients and clinicians:

1. *Transition between hospital and outpatient services*: admission to hospital; discharge and transition between hospital and outpatient treatment; transition between different outpatient settings.
2. *Continuity of care*: management/coordination of treatment; staff behaviour and skills; sharing of information between different professionals; provision of information to patients.
3. *Patients’ and carers’ involvement*: patients’ and carers’ involvement in treatment planning; how patients’ and carers’ preferences are reflected in the choice of specific settings, services and treatment options.
4. *Accessibility and availability*: appropriateness and timeliness of care provided; cooperation of mental health services with other healthcare services and with social services.

### Procedures

#### Establishing exposure to ‘specialisation’ or ‘continuity’ of care approach

To reflect differences in how mental healthcare services operate in the five countries, different procedures are used to ascertain exposure status to either a ‘specialisation’ or ‘personal continuity’ of care approach.

In the UK and in Italy, all patients admitted to the same hospital are treated under the same care approach, either a ‘specialisation’ or ‘personal continuity’ approach. In these countries, the local providers of care, that is, an ‘NHS Trust’ in UK and a ‘Dipartimento di salute mentale’ in Italy, may follow either the ‘specialisation’ or ‘personal continuity’ of care approach. The care is organised in the same way for all patients treated by the same provider of care which operates in a specific geographic area.

In Belgium, Germany and Poland, patients admitted to the same hospital can be treated under either a ‘specialisation’ or a ‘personal continuity’ of care approach. This is determined by patients’ health insurance arrangements (Germany) and/or by a mental health team decision in which the patient may or may not be involved (Belgium, Poland and Germany). Therefore, a patient is included in either group based on the primary hospital clinician report at discharge in Belgium, Poland and Germany.

#### Recruitment, obtaining informed consent and baseline interviews

Patients consecutively admitted to participating hospitals in each country from 1 October 2014 to 31 December 2015 are screened within two days of admission and recruited in the study. Patients will be followed-up for 1 year following their entry into the study. The end of follow-up is planned by 31 December 2016.

An internal pilot of study procedures was conducted from July 2014 to September 2014 and showed that study procedures are feasible.

First contact between the patient and the research staff takes place within two working days from the hospital admission to minimise potential selection bias due to early discharge of some patients, and to standardise the period of patient experience in the hospital. However, to ensure that as many eligible patients as possible are recruited and to increase the representativeness of the sample, there is some flexibility in recruitment. The initial contact can be postponed until the discharge if the patient wishes, or he/she is deemed to be too unwell by the clinician in charge. Before the patient is contacted by the researcher, a clinician will ask the patient for his/her assent to participate in research. If assent is obtained, the clinician introduces the researcher to the patient. The researchers explain the study and obtain written informed consent for participation in the study from the patients in face-to-face meetings. Any reason for which the interview is not conducted within two working days from the admission will be documented.

To enable an accurate estimation of selection bias, any pause in the recruitment (eg, for holidays or if all the researchers are on leave at the same time or for any other organisational reason) will be documented by specifying each day of recruitment and non-recruitment.

**Figure 3:**
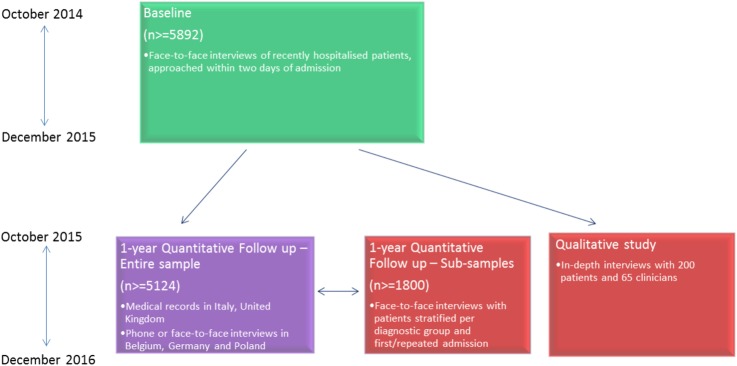
COFI recruitment and data collection plan. COFI, Comparing policy, framework, structure, effectiveness and cost-effectiveness of functional and integrated systems of mental healthcare.

The overall COFI recruitment and data collection plan is reported in figure 3.

#### Data collection at one-year follow-up

Clinical outcomes, employment, accommodation, living status, friendships, untoward events and indicators of care costs will be followed up for the *entire sample* via *medical records or telephone contact*.

To assess patients’ subjective quality of life, global social contacts, experienced and anticipated discrimination, perceived socioeconomic status, satisfaction with care, contacts with primary and secondary care professionals, and for patients’ experienced continuity of care we will randomly select a *subsample of patients, who will be interviewed face-to-face*.

Patients and clinicians interviewed for the *qualitative study* will be purposively selected from among those included in the entire sample. Selection criteria are reported in the following paragraph.

### Sample size determination

The sample size for the quantitative study was calculated with the aim to detect a 5% difference in rates of re-admissions (ie proportion of patients re-hospitalised during the one year follow up) between the two care approaches from 27.5% to 32.5% with 80% power at the 5% significance level.

The sample size required for an individual-level comparison not considering clustering of outcomes within centres and hospital would be 2716 patients.

We calculated an average cluster size per hospital of 150 patients recruited over one year. Taking into account a 15% drop out rate, the average number per hospital of patients recruited and followed up will be 128. The variance inflation factor (or design effect, IF) is given by IF=1+(m−1)×ρ; where m=cluster size, and ρ=ICC (inter-cluster correlation coefficient). The ICC in a cluster with such a large number of patients is expected to be very small.^37^ With m=128 and ρ=0.007, IF is 1.90, and the adjusted sample size is N=5162 (1034 per country), requiring eight hospitals per country with an average number of 150 patients recruited per hospital, ie 1200 patients per country and 6000 overall.

We will carry out follow-up face-to-face interviews with a subsample of patients who will be 18–65 years of age. The patients will be stratified based on diagnostic group (ICD codes: F20–29, F30–39, F40–49)^27^ and on whether the index admission was their first one or they have had previous hospital admissions. We will aim to interview 30 patients per stratum, resulting in 360 patients per country (60×6 clusters) and an overall sample of 1800 patients (360×5 countries).

For the in-depth interviews, we will purposively select 40 patients and 13 clinicians in each country, resulting in a total sample of 200 patients and 65 clinicians.

Within the sample, there will be a mix of: (1) patients treated with ‘specialisation’ and ‘personal continuity’ of care approaches; (2) those who were rehospitalised during the follow-up and those who were not; and (3) patients with different diagnoses (F20–29, F30–39, F40–49).

Clinicians will be included taking into account: (1) the care approach (‘specialisation’ or ‘personal continuity’) adopted by the service they work within and (2) their qualification: psychiatrists, psychologists, nurses/social workers.

## Data analysis plan

### Adjustment for confounders

The coexistence of the two care approaches in the five participating countries may be considered a major strength of the present study. Yet, clustering in outcomes may be found within hospitals and countries; therefore, we will explore random effects for hospitals as part of mixed regression models.

### Analysis of clinical and social outcomes

Mixed effects models with an unstructured variance matrix will be used to assess differences in the primary outcome (rehospitalisation), and in the other clinical and social outcomes between the two groups. We will explore random effects for the hospitals or ‘specialisation’ and ‘personal continuity’ of care clusters within the hospital (in Germany, Belgium and Poland), and use fixed effects for the following covariates:

- Age, gender, diagnostic group, whether or not a patient has been previously admitted, severity of illness at baseline, social situation (SIX score) and formal status of the patient at baseline.

We will check the selection bias by comparing data of our sample with the aggregate data for the total group of admitted patients during the same period.

Potential mediators of effectiveness may be: (1) the intensity of treatment, that is, sum of inpatient days and outpatient visits; (2) the experience of hospital care within the index admission, which has been shown to be predictive of better outcomes following discharge.^38^ ^39^ A higher intensity of treatment or a better experience of hospital care may occur in either the ‘specialisation’ or the ‘personal continuity’ of care approach and be responsible for better outcomes.^21^ ^38^ We will develop a mediation model^40^ in order to identify and explicate a potential effect of intensity of treatment and/or experience of hospital care during the index admission for assessing the effectiveness of the two approaches.^41^

Exploratory analyses to compare clinical outcomes, social outcomes and untoward events between the ‘specialisation’ and ‘personal continuity’ of care approaches will also be carried out on subgroups of patients defined by:

- Age (younger or older than 40 years);
- Gender (males or females);
- Primary psychiatric diagnosis (F20–29 or F30–39 or F40–49);
- First or repeated admission;
- Socioeconomic situation (receiving state benefits due to low income or not);
- Migrant status (ie, country of residence different from country of birth or not);
- Presence of physical comorbidity/comorbidities for substance use, diabetes, cardiovascular diseases or not.

All subgroup analyses will be performed by including the relevant variables as interaction terms in the models.

### Analysis of costs

The economic analysis will be led by the Centre for the Economics of Mental and Physical Health at King’s College London. Unit costs for each service measured with the CSRI will be calculated for each country. In order to compare costs across countries with different currencies, purchasing-power parities will be used to transform the country-specific costs into a single currency, thus allowing for analysis of the total sample as well as cross-national comparisons. The likely different impact of cost externalities or different cost structure of the two care approaches will be evaluated. Multiple regression will be used to adjust for the baseline patient characteristics in all tests of differences in costs and clinical outcomes. Cost-effectiveness will be then explored through the calculation of incremental cost-effectiveness ratios (ICER), defined as the difference in mean costs divided by difference in mean effects.

### Analysis of experience of care

The analysis of patient satisfaction with care and perceived continuity of care measured through quantitative methods will follow similar procedures to those described in the ‘analysis of clinical and social outcomes’ paragraph.

Data from semistructured interviews with patients and clinicians will be audio taped and transcribed verbatim, ensuring the removal of any identifying information to maintain anonymity. Transcripts will be analysed through a thematic content analysis method.^42^ Study centres in each country will generate a list of emerging codes based on a line-by-line analysis of the first three interviews with patients and first two with clinicians in the respective country.^43^ Consistency of coding will be assessed across all centres. First, researchers at the Institute of Psychiatry and Neurology of Warsaw (IPIN) and at Queen Mary University of London (QMUL) will screen the databases containing the coding results of the first interviews in each centre and discuss any discrepancies with the relevant centre. Second, researchers from all centres will provide further verification and clarification of the meanings of the codes during a one-day workshop. Third, each partner will code two interviews with patients and two interviews with clinicians (conducted in English or translated into English), and researchers at IPIN and QMUL will assess the coded data for discrepancies. Separate codebooks will be developed for interviews with patients and with clinicians, and interviews will be coded based on these.

Codes will be categorised based on their English translations. To obtain meaningful themes, the emerging categories and codes will be organised and grouped. Researchers from all centres will verify the emerging categories and themes to ensure consistency across the data set. Descriptive counts of themes, categories and codes will be use to summarise the data set.

QMUL group is experienced in conducting qualitative analyses of data from different countries as they have already led previous similar studies in which IPIN was also involved.^44^ ^45^

### Secondary analysis based on continuity of care as a ‘continuum’

A wide variability of care arrangements may support specialisation of clinicians and teams or personal continuity of care. This may determine intragroup differences among the services included within the categories of ‘specialisation’ and ‘personal continuity’ of care approaches. For example, the presence of a ‘care coordinator’ may guarantee continuity of care even when there are two different primary clinicians in the hospital and in the community. On the other hand, even when patients have the same primary clinician in the hospital and in the community, they may be treated for short periods of time by ‘specialist services’ such as home-treatment teams, emergency wards or psychiatric intensive care units led by different primary clinicians.

We will investigate the variety of system characteristics that lead to the implementation of specialisation and personal continuity of care in the five participating countries. This part of the study will focus on the financial and healthcare governance mechanisms that support both care approaches and will be led by the University of Louvain.

To explore this in more depth, we will also develop an instrument assessing continuity of care as a continuum. We will use this instrument to identify and rate different care arrangements within the two alternative approaches.

The association between different levels of continuity of care and the outcomes will be tested through mixed models taking into account random effects at an hospital level and fixed effects of the same covariates described above for the primary analysis.

## Discussion

COFI is a natural experiment which will overcome limitations of the currently available evidence:

- It is a multicentre study carried out in countries with very different traditions, funding mechanisms and policies for mental healthcare. In all these countries, care approaches supporting either specialisation or personal continuity of care coexist. This will allow for comparisons to be made within a similar context and not be confounded by country-specific characteristics. The service arrangements supporting specialisation or personal continuity of care may vary substantially among the countries, which will make our findings more robust and increase their generalisability to other countries and contexts.
- It is powered to detect very small differences in clinical and social outcomes between the two care approaches assessed that are relevant from a policy decision-making point of view (eg, 5% in rates of rehospitalisation).
- A comprehensive range of outcomes will be assessed, encompassing clinical outcomes, social outcomes, safety of care indicators, patient satisfaction with services and cost-effectiveness.
- Care approaches supporting specialisation or personal continuity of care which are assessed in this study are already established as routine services and are not just pilot experimental services. This will be a methodological safeguard from the ‘novelty bias’ found in the existing literature in which the newly established approaches (either supporting specialisation or continuity of care) tend to have better outcomes than the traditional ones.^21^

### Advantages of the adopted methodology

COFI will be the first natural experiment to compare different approaches to routine mental healthcare provision.

Natural experiments have a number of advantages over randomised controlled trials in health service research.^46–49^

From a practical point of view, the costs related to reorganisation of the mental healthcare services in multiple areas of a country for an experimental trial would not be sustainable or feasible. With a natural experiment design, a high number of patients can be recruited and the effectiveness of different care approaches can be assessed in different areas with no excess treatment costs.

From a scientific point of view, an assessment of routinely provided care avoids the ‘novelty bias’ related to the enthusiasm of patients and clinicians for a newly established way of receiving or providing care.^21^ Patients will be receiving and evaluating standard care instead of a new intervention.

The choice of the sites is very important for a natural experiment. The clinical sites recruited reflected a wide range of geographical areas. The five participating countries have both care approaches as part of their routine mental healthcare provision in different cities or areas of a different city; therefore, the comparison will not be biased by within-country variables. Potential clustering of outcomes will be explored considering random effects at a hospital level.

The countries selected for COFI offer a wide variety of traditions, policy models and funding mechanisms of mental healthcare. They are located in different areas of Europe and share commonalities in sociopolitical characteristics as well as practice and organisation of mental healthcare with non-included neighbouring countries. Therefore, it is expected that COFI findings can be generalisable to a larger number of countries in Europe than those included in the study.

### Potential challenges

Patients will be recruited during the first days of hospital admission. Many patients may not be able to give consent or, even if they are, be unwilling to participate in the study because they are severely distressed. A number of patients admitted during the weekend and discharged after a few days may be difficult to contact. In order to enable researchers to promptly screen all admitted patients, we have established close links with the hospital staff. Presentations/refreshers on the study at all sites are carried out to make sure that all the clinical staff in the hospitals are informed on the study and information on new admissions is quickly passed on to the researchers.

One important challenge for the follow-up relates to the different ways in which medical records are stored in different countries. In UK and Italy, continuous electronic medical records, including information on a given patient within and outside the hospital, are available; however, this is not the case in other countries. Therefore, patients will receive short phone interviews at follow-up in Germany, Belgium and Poland. This may determine attrition rates higher than 15% for the primary outcome in these countries; in UK and in Italy, attrition rates are likely to be lower than 15%. The potential imbalance in missing data at follow-up among countries will be controlled in the mixed model adjusting for fixed effect of baseline patient-level variables and random effects at a hospital level.

### Expected outputs

The dissemination of COFI results will not only follow the usual pathway of publications in scientific journals but also take advantage of electronic means of communication (newsletters, twitter, website and online journals).

Moreover, to facilitate the translation of findings into national mental healthcare policies and practices, we will: (1) produce recommendations for policy decision-making based on study findings; (2) organise workshops inviting all relevant stakeholders.

Recommendations on policy decision-making will be circulated to different stakeholders: (1) national governments; (2) professional associations; (3) carers and users groups; (4) opinion leaders, managers and policymakers at a national level. The stakeholders will be able to familiarise with study findings in order to make their contribution in translating research evidence from COFI into the practice of policy decision-making.

The organisation of national workshops in all the participating countries and of one European workshop to discuss COFI findings will allow direct contact with stakeholders. Such events will offer opportunities for feedback, as well as for the creation of strong relationships with stakeholders and interest groups that will enhance the impact of study findings on national mental health policies.

## Current study status

In June 2015, COFI is in the ninth recruitment month (out of 15 months). During the study period, 13 916 patients were admitted in the included hospitals. Among them, 8153 were eligible for the study (58.6%); 4484 of the eligible patients gave informed consent to participate in the study (55%). Eligibility and opt-in rates are similar to previous studies on hospitalised patients^38^ ^50^ and the current recruitment rate is in line with the planned recruitment target (6000).
